# Oncologists’ Satisfaction with Virtual Care: A Questionnaire

**DOI:** 10.3390/curroncol31060248

**Published:** 2024-06-05

**Authors:** Amaris Karin Balitsky, Nathan Cantor, Karen Zhang, Greg Pond, Mark Norman Levine

**Affiliations:** 1 Hamilton Health Sciences, Juravinski Cancer Center, Hamilton, ON L8V 5C2, Canada; 2 Escarpment Cancer Research Institute, Hamilton, ON L8V 5C2, Canada; 3 Department of Oncology, McMaster University, Hamilton, ON L8V 5C2, Canada; 4 Michael G. DeGroote School of Medicine, McMaster University, Hamilton, ON L8S 4L8, Canada; 5 Department of Psychiatry and Behavioural Neurosciences, McMaster University, Hamilton, ON L8S 4L8, Canada

**Keywords:** virtual care, oncology, clinician

## Abstract

Introduction: Although virtual care (VC) has become an integral part of oncology care and healthcare delivery, clinicians’ perspectives on and satisfaction with this modality are not well understood. Methods: Using a National Network Forum framework and expert panel review, we developed a questionnaire to measure oncologists’ satisfaction with VC. The questionnaire was distributed to Canadian oncologists through medical society email lists (n = 1541). We used a 5-point Likert scale to capture their responses, which included strongly disagree (1), disagree (2), undecided (3), agree (4), and strongly agree (5). Results: A total of 61 oncologists and/or oncology trainees, of 768 (7.9%) who opened their email, completed questionnaires between October 2022 and January 2023. Every questionnaire item had a response rate greater than 98%. Seventy-two percent of the respondents were satisfied with VC. Oncologists who were less comfortable with technology were more likely to report lower levels of satisfaction (*p* < 0.001, Wilcoxon rank-sum). The questionnaire items that received the highest levels of agreement were related to VC reducing costs and improving access for patients and concerns about missing a diagnosis and assessing patients’ functional status. The questionnaire items that received the greatest disagreement were related to VC improving access for patients with language barriers, VC being associated with time-savings for clinicians, improvements in clinical efficacy, and more readily available lab tests. Conclusions: Most of the oncologists surveyed are satisfied with VC; however, there are some concerns with VC that need to be addressed. Future research on optimizing VC should address clinicians’ concerns, in addition to addressing the patient experience.

## 1. Introduction

Virtual care (VC) is the remote interaction between clinicians and patients. The goal of VC is to promote high-quality and effective patient care [1]. Except for the use of telehealth in remote settings, there was little adoption of VC prior to the COVID-19 pandemic. By the second quarter of 2020, the proportion of all ambulatory visits that took place virtually in Ontario was 70.6%, compared to only 1.6% in 2019 [2]. Similar changes were observed in the United States [3]. An abrupt pivot to VC occurred in oncology clinics [4,5,6], and patients with cancer have continued to receive VC since the pandemic restrictions have been eased [7].

Assessing the effectiveness of VC requires an understanding of both patient and clinician experiences. From the patient perspective, there is evidence that VC as an adjunct to the standard of care improves health-related quality of life [8,9]. Other studies suggest high levels of patient satisfaction [10] and reduced financial costs [11] are associated with VC. Evidence on VC directly compared to in-person care is limited [12].

There are limited data on oncologist-specific experiences and satisfaction with VC. Understanding physicians’ perspectives surrounding VC is important, as the interaction between patients and their providers is bidirectional. A mixed-methods study of radiation oncologists found high rates of VC uptake during the early height of the pandemic, with over half of them reporting that VC resulted in an improvement or no difference in the clinical experience [13]. They identified physical examination and building personal connections with patients as limitations to VC [13]. Some studies have described differences in the perception of VC between cancer patients and oncologists [4,14]. Compared to patients, physicians are more likely to report concerns regarding the safety and quality of care [4], care coordination, and time spent with patients [14]. The impact of these discordances between patients’ and physicians’ perceptions on cancer care is unclear, but it highlights the need to evaluate oncologists’ experiences with VC in a systematic manner.

To our knowledge, there are no oncologist-specific questionnaires measuring physician satisfaction with VC. To address this gap, we developed an oncologist-specific questionnaire to understand oncologists’ experiences and satisfaction with VC. We then distributed our questionnaire to a sample of Canadian oncologists to measure the oncologists’ satisfaction with VC and its related modifiers.

## 2. Methods

### 2.1. Study Design

We developed a questionnaire to conduct a prospective cohort study design.

### 2.2. Questionnaire Development

We developed a questionnaire utilizing a literature review and guidance from an existing framework (National Quality Forum [15]). The authors A.B., K.Z., and M.L. first generated an inclusive list of 38 items (or questions) based on the following domains: clinical effectiveness, patient experience, access to care and financial cost/impact on patients, and other themes identified from previous qualitative work on oncologists’ perceptions of telehealth video care [16]. An expert panel of 11 individuals from the Juravinski Hospital and Cancer Centre, a large cancer centre in Ontario, Canada, including psychologists, nurse practitioners, medical oncologists, hematologist oncologists, and radiation oncologists, reviewed each questionnaire item for face validity. We asked the panel to rate each item twice based on its relevance to the construct of oncologist satisfaction and the clarity of the question. To limit bias from the expert panel, we did not group items into specific domains. We scaled items using a “summated ratings” methodology [17]. We used a 5-point Likert scale to capture their responses, which included strongly disagree (1), disagree (2), undecided (3), agree (4), and strongly agree (5). The inclusion of “undecided” allowed us to maintain a balance of positive and negative responses. Items with a mean score of 3 or less for relevance or clarity were considered for elimination or a rewrite, respectively. Experts were encouraged to write additional comments about the questionnaire items, for example, suggesting any important dimensions of patient satisfaction that were missing. The authors A.B. and K.Z. reviewed all answers independently, and any disagreements were resolved by consensus. Two items were eliminated based on their lack of relevance, and three items were added based on suggestions from the expert panel. Four items were re-worded based on their lack of clarity. At the end of the process, 39 items were included in the final version. See the Supplemental Materials for a complete item list.

### 2.3. Data Collection

In October 2022, using a modified Dillman approach [18], we sent emails to Canadian professional societies’ email servers containing an invitation to participate in this study with an electronic link to our questionnaire. The three Canadian professional societies’ included; (1) the Canadian Association of Medical Oncologists; (2) the Canadian Hematology Society; and (3) the Canadian Association of Radiation Oncology. The email with the questionnaire link was sent out multiple times. The aim was to obtain feedback from clinicians across Canada. This study was approved by the Hamilton Integrated Research Ethics Board. The participants read a consent statement prior to participating in the questionnaire.

All the questionnaire and demographic data were captured anonymously within REDCap software (Version code 14.0.30), a firewall-protected database. We collected the following demographic information: age, sex, subspecialty (medical oncology, malignant hematology, or radiation oncology), and years in practice (trainee, <5 years, 5–10 years, or >10 years). We also collected data related to the participants’ comfort with technology (rated on a Likert scale; 1 = not comfortable, 10 = very comfortable) and proportion of practice comprising VC (<25%, 25–50%, >50%). Comfort with technology was dichotomized, with ≥7 out of 10 considered more comfortable with technology and <7 considered less comfortable (based on being less than and greater than the 25th percentile).

### 2.4. Statistical Analysis

Descriptive statistics were used to summarize the questionnaire response rates and baseline demographics. Comparisons between groups was carried out using Wilcoxon rank-sum tests due to the ordinal nature of the data. We calculated Cronbach’s alpha to measure the internal consistency (correlations between the items comprising the scale). We measured the construct validity of the questionnaire via Spearman’s rank correlation. We performed statistical analyses in STATA 16.1 (College Station, TX, USA). In the event of missing data, we planned to perform multiple imputation analyses. All tests were two-sided, and statistical significance was defined as the α = 0.05 level, with no adjustments for multiple testing.

## 3. Results

The participants completed the questionnaires between October 2022 and January 2023. Based on the sum of each society’s largest number of recipients from any one questionnaire request, 1541 individuals potentially received the email, and 768 individuals opened the email. Sixty-one oncologists and/or oncology trainees responded to our survey (response rate = 7.9%), and every questionnaire item had a response rate greater than 98%. The response rates varied by professional society (3–12%). The participants’ specialties included radiation oncology (49%), medical/hematology oncology (41%), and trainees (10%). Fifty-three percent of the participants had been in practice >10 years. See Table 1 for a detailed breakdown of their demographic information.

**Figure 1:**
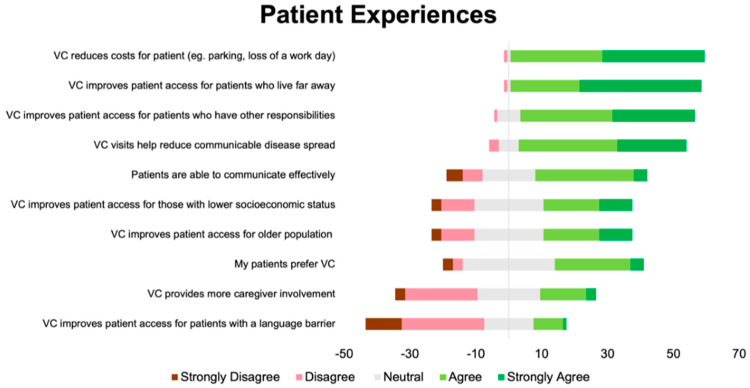
Diverging stacked bar plot showing participants’ responses to items related to patient experiences. Green indicates positive response (4 or 5 out of 5), grey indicates neutral response (3 out of 5), and red indicates a negative response (1 or 2 out of 5).

**Figure 2:**
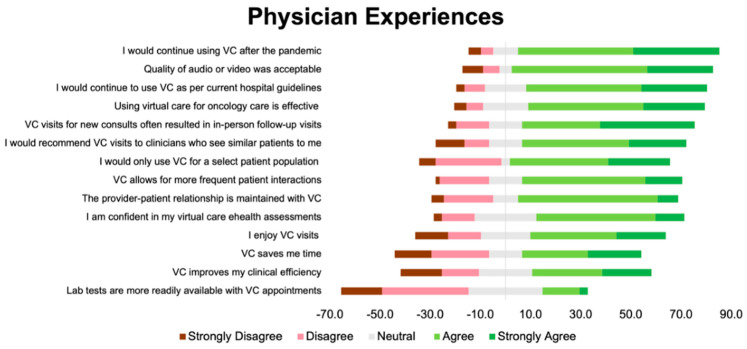
Diverging stacked bar plot showing participants’ responses to items related to physician experiences. Green indicates positive response (4 or 5 out of 5), grey indicates neutral response (3 out of 5), and red indicates a negative response (1 or 2 out of 5).

**Figure 3:**
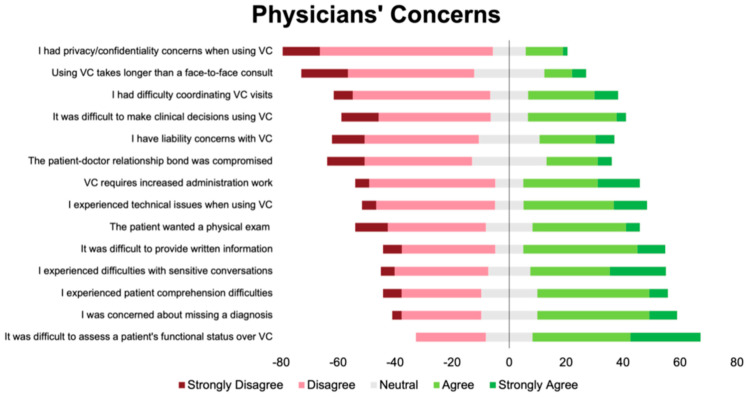
Diverging stacked bar plot showing participants’ responses to items related to physician concerns. These items were answered in reverse ordinal order. Green indicates positive response (4 or 5 out of 5), grey indicates neutral response (3 out of 5), and red indicates a negative response (1 or 2 out of 5).

Overall, 72% of the respondents agreed or strongly agreed that they were satisfied with VC. The questionnaire items with the percentages of respondents in agreement or strong agreement with each item (or disagreement or strong disagreement for flipped items) are shown in Table S1 of the Supplementary Materials. Fifty-four percent of the respondents agreed or strongly agreed that they enjoyed VC. Over half of the participants (54%) did not agree or were neutral about whether patients preferred VC. Prior to the analysis, we grouped related items into the following categories—(1) Factors related to oncologists’ perceptions of patient experiences; (2) factors related to physician experiences; and (3) factors related to physician concerns—in reverse ordinal order (Figure 1, Figure 2 and Figure 3).

The questionnaire items that received the most agreement were VC reduces costs and improves access for patients, physicians would continue VC after the pandemic and/or as per hospital guidelines, and concerns about missing a diagnosis and assessing patients’ functional status with VC. The questionnaire items that received the most disagreement were VC improves access for patients with language barriers, lab results are more readily available, there are privacy concerns when using VC, and VC takes longer compared to in-person visits. The answers were split for the following items: VC is associated with time-savings for the clinician, and VC takes longer compared to in-person visits.

In exploratory analyses, we compared the overall oncologist satisfaction with VC according to the following factors, age, years of practice, comfort with technology, and specialty, using Wilcoxon rank-sum tests. The median levels of satisfaction were comparable by years of practice (≤10 vs. >10 years), but physicians less comfortable with technology were more likely to report lower levels of satisfaction with VC compared to those who were more comfortable (*p* < 0.001, Wilcoxon rank-sum). Given the 98% response rate, multiple imputation analysis was not performed.

Overall, the questionnaire had good internal consistency (Cronbach’s α = 0.95, average inter-item correlation = 0.37) and construct validity (Spearman’s rank correlation = 0.89).

## 4. Discussion

The present study uniquely developed a 39-item questionnaire that measures oncologists’ satisfaction with VC. Overall, 72% of the participants surveyed are satisfied with VC. However, it should be kept in mind that the response rate was quite low and that this cohort may not be representative of all oncologists. Although the majority of participants are satisfied with VC, there are areas of concern that need to be addressed, such as concerns around missing a diagnosis and ability to assess functional status.

The overall high levels of physician satisfaction are consistent with other published survey studies of provider satisfaction with VC [4,14]. Many of the positive aspects of VC that the physicians identified were related to clinician perceived patient-specific factors (i.e., reduced costs, improved access). These factors may be particularly important to patients with cancer, as these patients often face significant financial costs, both with respect to out-of-pocket drug costs [19] and indirect costs, like travel expenses [11]. Surprisingly, from the clinician perspective, there were few concerns with using VC with elderly patients or concerns with disparities in socioeconomic status and accessibility. For some, VC allows patients to have their appointment from the comfort of their own home, without time spent travelling and waiting in labs and waiting rooms. Numerous studies have demonstrated improved patient satisfaction and quality of life with VC modalities of healthcare [8,9].

In our study, physician satisfaction was similar with respect to gender, years of practice, and oncology specialty. Physicians with lower levels of self-reported comfort with technology reported lower levels of satisfaction with VC. Our study suggests that physicians with lower levels of comfort with technology are less likely to be satisfied with VC, which could in turn influence the frequency of their VC practice and the clinical efficacy of VC. That said, in a study that favoured the use of electronic patient-reported symptoms compared to standard oncology care, participants were trained on the technology before its implementation, and individuals with less computer experience faired equally well [20]. In this current cohort, however, physicians rapidly adopted VC, with little to no training. Appropriate technology training may improve clinician satisfaction with new modalities of VC. Studies focusing on electronic symptom monitoring or other platforms should consider evaluating physicians’ technological comfort levels as a potential barrier to (or facilitator of) their interventions.

Privacy concerns, often raised in the context of telemedicine [21], were not identified as a prominent issue by the physicians in our study. Virtual platforms introduce potential vulnerabilities such as data breaches, unauthorized access, and the risk of patient information being intercepted during transmission. Although not identified as a concern in this study, future studies on virtual care should ensure the confidentiality and security of patients’ sensitive medical information.

Concerns about missing a diagnosis and difficulties with assessing patients’ functional status were among the most negative aspects of VC identified. It is possible that the physicians had more difficulty with assessing functional status in this cohort given the fact that the majority of VC took place over the phone without video observation. Patients’ functional status, including activities of daily living and performance status, is an essential consideration in oncology treatment decisions, including eligibility for treatment. Prior to and during the pandemic, feasibility studies demonstrated high levels of completion rates for and patient acceptance of certain functional assessments via telehealth, such as comprehensive geriatric assessments [22]. More research is needed on whether these virtual functional status assessments are accurate compared to in-person evaluations and share similar predictive value surrounding the treatment responses and toxicities. Concerns about missing diagnoses are also valid given the complex nature of patients with cancer. Integrating a remote patient monitoring approach to monitoring patient symptoms and vital signs could address concerns about the lack of physical exams.

The answers were split on whether VC was perceived to save the clinicians’ time and increase efficiency. This divide on the perception of whether VC saves time and increases efficiency may relate to differences in institutional electronic medical record capabilities or institutional differences in administration practice.

## 5. Strengths and Limitations

This study has some strengths. First, we created a questionnaire specifically tailored to assessing oncologists’ perceptions using a pre-existing framework and expert consensus. Second, we included a variety of oncologists from different specialties (hematology, medical oncology, and radiation oncology) and trainees. Third, a rigorous process was performed to identify relevant questions and create the questionnaire. This study also has some key limitations. The questionnaire was distributed through medical societies’ email lists. This design limited our ability to directly question individuals prospectively. Second, we had low response rates for the questionnaire despite using a gold-standard methodology [18] to increase response rates and incentives. In some cases, we were limited by society-specific email policies (i.e., one society had a policy that only one email could be sent). In addition, one of the societies included benign and malignant hematologists, diluting the sample further. Third, the low number of responses limited our ability to perform exploratory factor analysis and generate specific domains. A future larger validation study could address these limitations. Lastly, we only sent our questionnaire to physicians, and therefore our results may not generalize to other clinicians involved in oncology patient care, such as nurse practitioners, physician assistants, and psychologists. Although early research suggests similarly high levels of satisfaction with VC among other allied healthcare professionals [23], further studies should examine a wider range of care providers.

## 6. Conclusions

VC has become an integral part of oncology care and healthcare delivery at large. While the focus thus far has primarily been on the benefits VC offers to patients, our study sheds light on clinician satisfaction with VC and areas of discomfort and uncertainty. Moving forward, it is important that research efforts concentrate not only on optimizing VC for patients but also on addressing the concerns of those delivering the care. By continually refining and enhancing VC through collaborative research and innovation, we can ensure accessible and high-quality healthcare to all patients living with cancer.

## Figures and Tables

**Table 1:** Demographic information of study participants.

Demographic Characteristic	Proportion (n (%))
**Gender**	
Female	27 (44)
Male	33 (54)
Other	1 (2)
**Age**	
<40	24 (39%)
40–60	29 (48%)
>60	8 (13%)
**Oncology Subspeciality**	
Radiation Oncology	30 (49)
Medical Oncology	21 (34)
Hematology Oncology	4 (7)
Trainee	6 (10)
**Years in Practice**	
<5 Years	19 (31)
5–10 Years	10 (16)
>10 Years	32 (53)
**Type of Virtual Care**	
Phone Only	22 (36)
Phone and Video	38 (62)
Video Only	1 (2)
**Proportion of Practice Consisting of VC**	
<25%	26 (43)
25–50%	26 (43)
>50%	8 (13)
Not Reported	1 (2)
**Comfort with Technology**	
<7 out of 10	8 (13)
≥7 out of 10	53 (87)

## Data Availability

The data are stored in a REDCap database and can be made available upon request.

## Supplementary Materials

The following supporting information can be downloaded at https://www.mdpi.com/article/10.3390/curroncol31060248/s1. Table S1. List of questionnaire items with number of responses indicating agreement or strong agreement with the individual item.
