# Supplementary material for: Oncologists’ Satisfaction with Virtual Care: A Questionnaire
Source: Curr Oncol. 2024 Jun 5;31(6):3269–77. doi: 10.3390/curroncol31060248 (PMC11202864; doi:10.3390/curroncol31060248)
Supplement: Supplementary file 1 [file curroncol-31-00248-s001.zip › curroncol-2988573-supplementary.pdf]

Supplemental material

Table S1. List of questionnaire items with number of responses indicating agreement or strong agreement with the individual item.

Questionnaire Item	*Agree or Strongly Agree, N (%)*
1. *I would only use virtual care for a select patient population (post-pandemic)*	39 (64%)
2. *Using virtual care for oncology care is effective*	43 (70%)
3. *I would continue to use virtual care as per current hospital guidelines (post pandemic)*	44 (72%)
4. *Virtual care allows for more frequent patient interactions*	39 (64%)
5. *Virtual care visits for new consults often resulted in in-person follow-up visits*	42 (69%)
6. *Lab tests are more readily available with virtual care appointments*	11 (18%)
7. *Virtual care visits help reduce communicable disease spread*	51 (84%)
8. *I am confident in my virtual care e-health assessments*	36 (59%)
9. *Virtual care provides more caregiver involvement*	17 (28%)
10. *Patients are able to communicate effectively*	34 (56%)
11. *My patients prefer virtual care*	27 (44%)
12. *The provider-patient relationship is maintained with virtual care*	39 (64%)
13. *Virtual care improves patient access for patients who live far away*	58 (95%)
14. *Virtual care improves patient access for patients who have other responsibilities (e.g. elder parents, young kids)*	53 (87%)
15. *Virtual care improves patient access for patients with a language barrier*	10 (16%)
16. *Virtual care improves patient access for older population*	38 (62%)
17. *Virtual care improves patient access for those with lower socioeconomic status*	27 (44%)
18. *Virtual care reduces costs for patient (e.g. parking, loss of a work day)*	59 (97%)
19. *Virtual care saves me time*	29 (48%)

20. I enjoy virtual care visits	33 (54%)
21. I would continue using virtual care after the pandemic	49 (80%)
22. Quality of audio or video was acceptable	49 (80%)
23. Virtual care improves my clinical efficiency	29 (48%)
24. I would recommend virtual care visits to clinicians who see similar patients to me	40 (66%)
25. It was difficult to make clinical decisions using virtual care	32 (53%) *
26. It was difficult to provide written information	24 (39%) *
27. Using virtual care takes longer than a face-to-face consult	37 (61%) *
28. I experienced patient comprehension difficulties	21 (34%) *
29. I experienced difficulties with sensitive conversations	23 (38%) *
30. The patient wanted a physical exam	28 (46%) *
31. The patient-doctor relationship bond was compromised	31 (51%) *
32. I have liability concerns with virtual care	32 (53%) *
33. I was concerned about missing a diagnosis	19 (31%) *
34. Virtual care requires increased administration work	30 (49%) *
35. I had difficulty coordinating virtual care visits	33 (54%) *
36. I had privacy/confidentiality concerns when using virtual care	45 (74%) *
37. I experienced technical issues when using virtual care (e.g. internet connection)	28 (43%) *
38. It was difficult to assess a patient's functional status over virtual care	15 (25%) *
39. Overall, I am satisfied with virtual care	44 (72%)

* Indicates proportion in disagreement or strong disagreement. Disagreement or strong disagreement generally indicates more positive experiences with virtual care.
